# DDoS Attack Prevention for Internet of Thing Devices Using Ethereum Blockchain Technology

**DOI:** 10.3390/s22186806

**Published:** 2022-09-08

**Authors:** Rahmeh Fawaz Ibrahim, Qasem Abu Al-Haija, Ashraf Ahmad

**Affiliations:** Department of Computer Science/Cybersecurity, Princess Sumaya University for Technology (PSUT), Amman 11941, Jordan

**Keywords:** blockchain, Ethereum, smart contract, authorization, DDoS attacks, IoT

## Abstract

The Internet of Things (IoT) has widely expanded due to its advantages in enhancing the business, industrial, and social ecosystems. Nevertheless, IoT infrastructure is susceptible to several cyber-attacks due to the endpoint devices’ restrictions in computation, storage, and communication capacity. As such, distributed denial-of-service (DDoS) attacks pose a serious threat to the security of the IoT. Attackers can easily utilize IoT devices as part of botnets to launch DDoS attacks by taking advantage of their flaws. This paper proposes an Ethereum blockchain model to detect and prevent DDoS attacks against IoT systems. Additionally, the proposed system can be used to resolve the single points of failure (dependencies on third parties) and privacy and security in IoT systems. First, we propose implementing a decentralized platform in place of current centralized system solutions to prevent DDoS attacks on IoT devices at the application layer by authenticating and verifying these devices. Second, we suggest tracing and recording the IP address of malicious devices inside the blockchain to prevent them from connecting and communicating with the IoT networks. The system performance has been evaluated by performing 100 experiments to evaluate the time taken by the authentication process. The proposed system highlights two messages with a time of 0.012 ms: the first is the request transmitted from the IoT follower device to join the blockchain, and the second is the blockchain response. The experimental evaluation demonstrated the superiority of our system because there are fewer I/O operations in the proposed system than in other related works, and thus it runs substantially faster.

## 1. Introduction

The Internet of Things (IoT) permeates every facet of daily life worldwide. It was reported that the number of connected devices is projected to jump from approximately 27 billion in 2017 to 125 billion in 2030, an average annual increment of 12% [1]. People are progressively installing IoT devices in their homes as they become more common, such as smart TVs, Internet boxes, heating systems, home remote controls, lighting systems, etc. Robots and other intelligent devices working together increase the efficiency of automation systems in various industrial settings, including factories [2].

The IoT is now widely used in various industries, including healthcare, education, agriculture, and smart cities [3]. These use cases were just the start of the IoT’s development. The omnipresence of numerous objects, where they can interact and work together to deliver a variety of services, is the concept that underpins the Internet of Things and its many uses [4]. As a result, a huge selection of devices will be accessible. However, only authorized users are allowed access to the system. If not, it will be susceptible to various cyberattacks, such as distributed denial-of-service attacks (DDoS), in which numerous Internet of Things devices work together to send thousands of destructive requests to one central server, overloading it. The system is more susceptible to attacks like identity spoofing, message eavesdropping, message tampering, and other security issues because most communications take place wirelessly [5].

Additionally, different device types have constrained energy, memory, and computing resources, which makes it challenging for the IoT to be extensively embraced and deployed. IoT is frequently described as a system of systems, and many use-case scenarios only allow trusted users to access offered services. Because of this, every element of these ecosystems, including the devices, networks, and software applications, depends on well-established security principles like authentication, anonymity, and data integrity. However, the diversity of devices and resource constraints make traditional security solutions incompatible with such an ecosystem. Further raising costs is the common requirement for merging many security systems and solutions. A system with thousands of nodes can make it challenging to deploy centralized security solutions like Public Key Infrastructure (PKI) [6]. Lastly, each use case employs a security strategy, design, and deployment, which poses several difficulties when integrating new services and circumstances. New system security considerations must be offered as a result.

Unlike previous studies such as [7,8], which used private blockchain to authenticate IoT devices, the main distinguishing characteristic of our proposed system is the use of a public blockchain to authenticate IoT devices before communicating with the network. The main reason to select a public blockchain is to maintain advantages from blockchain properties like immutability, decentralization, and authenticity. Additionally, the proposed system uses blockchain to store only the IP addresses of legitimate devices, while, on the other hand, other studies such as [9] have a scalability problem since they store all data, regardless of whether it is malicious or benign. Moreover, unlike research studies that only employ blockchain technology as a record-keeping system [10], our proposed system utilizes blockchain technology as a first step to authenticate and verify IoT devices. Furthermore, the studies [11,12,13] do not mention in detail how the authentication process is accomplished, but, in our system, the authentication and communication processes are explained and implemented practically in detail.

In particular, in this study, newly popular technology is utilized to authenticate IoT devices on a network and to give a decentralized solution to prevent DDoS attacks on IoT devices. This kind of decentralized system is justified by data access being decentralized, and because that data, once placed in the chain, cannot be changed or removed. This is because changing or removing a block requires a sizable amount of computing power and is more expensive due to the cryptographic links that the blocks in the chain have with the blocks that came preceding them. The immutability attribute may be used to identify and monitor malicious IoT devices to prevent them from subsequently connecting to and interacting with IoT networks. Specifically, the following can be used to summarize this paper’s primary contributions:

- We use Ethereum blockchain technology to authenticate and validate these devices, which provides an authentic and tamper-proof platform to replace the present centralized system solutions and prevent DDoS attacks on IoT devices on the application layer.
- To prevent connecting to and communicating with IoT networks in the future, we track and store the IP addresses of malicious devices inside the blockchain.

The rest of this study is organized as follows: In Section 2, an explanation of the blockchain and the Ethereum chain type, a description of DDoS attacks, and an introduction to IoT technology are provided. In Section 3, the related work to this study is described. In Section 4, the methodology and processes that are used in this study are described and explained. Section 5 presents the findings, discussion, and comparison, as well as the performance evaluation. The paper is concluded in Section 6.

## 2. Background

Blockchain technology is used in many industries, including cryptocurrencies, healthcare, energy, digital documents, and IoT. The proposed system employs blockchain technology to prevent DDoS attacks on IoT devices in a decentralized manner without the help of third trusted parties.

**Figure 1:**
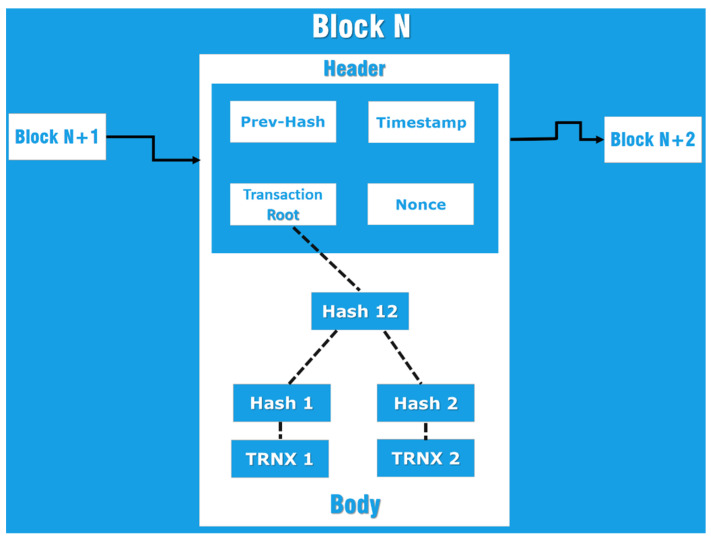
Block Structure [15].

In blockchain, all digital transactions are stored in a decentralized, open-source database. All transactions between all computers in the P2P network are created and shared using blockchain. Additionally, depending on the type of blockchain utilized, it enables all nodes in this network to apply a consensus technique called Proof of Work (PoW), Proof of Stake (PoS), or Proof of Authority (PoA) to verify the transactions entered in the block before they are added to the chain. The most significant attribute of blockchain is its decentralized nature, which eliminates the need for a third trusted party and makes it ideal to utilize in numerous applications such as energy, healthcare, and IoT technologies [14]. Each block in a blockchain has a header and a body, as seen in Figure 1.

In this proposed system, we use the Ethereum blockchain, which is a public blockchain that uses a cryptocurrency called Ether (ETH) for paying financial transactions and processing applications using smart contracts (SC) [8,16]. The SC created for the system controls network connectivity and verifies the IoT devices’ credentials before connection.

### DDoS Taxonomy

Distributed denial of service is a cyber-attack on a specific server or network; the purpose of DDoS is to disrupt the normal operation by flooding the targeted network resources such as IoT devices or servers with a constant flood of traffic, such as fraudulent requests, which overwhelms the system, causing a disruption or denial of service to legitimate traffic. Using IoT technology, an attacker may send malicious code to a house’s internet-connected webcam or home automation system [17].

Attackers attempt to attack susceptible devices by utilizing the default login and password lists on these devices and trial-and-error methods. Compared to computers and smartphones, IoT devices are more hackable due to poorly maintained firmware. The fact that they are always “online” makes them ideal for hackers to manipulate remotely. Once these devices have been infected, they are added to botnets and begin to overrun the server or service that has been targeted.

The Mirai botnet took down Twitter, Netflix, and other important websites in 2016, along with a sizable internet section. Additionally, it had an impact on the largest Russian banks as well as the entire country of Liberia [18]. Mirai targeted the DYN servers that manage internet traffic after infecting unsecured IoT devices like security cameras with malware [19]. The DDoS attack can be separated into application- and infrastructure-layer attacks [20,21].

***Application-layer attacks*** are those attacks that try to penetrate the IoT network infrastructure’s application layer, where packets are lost at a rate of one request per second (RPS) due to HTTP(Get/Post) requests saturating the application or web server, as well as other requests that target device applications like Windows, Apache, OpenBSD, and others. Because they generate traffic at a slower rate and the requests they send appear legitimate, these attacks are more challenging to identify and defeat because they start a back-end mechanism that disables services. Examples of these attacks include HTTP floods and DNS service-based assaults.

- HTTP flood attack: The cybercriminal in this attack uses legitimate HTTP GET or POST requests to launch a DDoS attack. These attacks do not use spoofing or reflection tactics, so they need less bandwidth to reach the targeted server than other attacks.
- DNS service attack: DNS flood is a distributed denial-of-service (DDoS) attack in which an attacker floods a domain’s DNS servers with requests to interrupt DNS resolution for that domain. A DNS flood attack can make it impossible for a server, API, or web application to respond to legitimate traffic because DNS resolution will be interrupted. Since the huge amount of traffic also arrives from various specific sites, querying for actual records on the domain and mimicking legal traffic, DNS flood attacks can be difficult to differentiate from regular heavy traffic.

***Infrastructure-layer attacks:*** By leveraging vulnerabilities in the transport or network layers of the IoT architecture, attackers of this type try to render the target device unavailable. They typically employ tactics of reflection or amplification to launch the attack. The attacker uses IP address spoofing to congest the victim’s network by reflecting the user’s submitted request as an unrequested reply. Making greater answers from smaller queries frequently leads to amplification, unnecessarily using bandwidth.

## 3. Related Work

In the last decade, several research studies have been conducted and proposed to mitigate and prevent DDoS attacks on IoT devices utilizing both centralized (traditional solutions) and decentralized solutions (blockchain-based solutions). In the following subsections, we review most of these studies.

### 3.1. Traditional Solutions

Different centralized solutions are proposed to solve the DDoS attack on IoT technology, such as in papers [22,23]. For instance, in [24], the authors proposed to mitigate the DDoS attack based on the cloud by increasing the capacity and taking the detection burden away from the attacked device by exporting flow records from edge routers and switches; those solutions have disadvantages because they need a third--party DDoS Protection Service (DPS) provider which implies a decrease in the performance and requires additional costs to deploy on the existing network because of its centralized nature.

Another centralized solution mentioned in [25] used honeypots and a central database to mitigate the DDoS attack. Honeypots are traps for intruders attempting to compromise the system’s security in this proposed technique. As the name implies, a honeypot attracts attackers to observe and analyze their method of initiating an attack by capturing information about the attacking agent, such as malware. The model depicts incoming server request anomalies using an intrusion detection system. If any such requests are discovered, they are directed to the honeypot rather than the main server. Using a honeypot, information about the suspect (who could be an attacker) is kept as logs in the database, including its IP address, MAC address, and other details. Following the collection of logs in the database, when an IDS detects a similar request, the information of the client request is compared to the stored log files. Based on the results, the main server sends a verification request to the client to verify its authenticity. If it is determined to be spam, the client is immediately stopped by the main server at the request stage. If the client is passed, the request moves on to the next stage, which is to be processed by the main server.

The Datagram Transport Layer Security (DTLS) protocol is one of the current Internet protocols that the authors of [26] use in this work to provide the first fully developed two-way authentication security solution for the Internet of Things. The suggested security system operates on top of common low-power communication stacks and is based on the most popular public key cryptography (RSA). They claim that depending on an established standard allows for the reuse of existing implementations, engineering methods, and security infrastructure, facilitating quick adoption of security.

This study [27], suggests an ECC-based user authentication protocol that manages IoT devices’ processing and data storage limitations. According to the analysis of their system security, the ECC-based protocol is suited for applications with higher security needs.

### 3.2. Blockchain Technology Solutions

It is an original and unique idea to use public key cryptography and digital signatures with the blockchain [28]. The most important security requirements, like transaction integrity, non-forgery, effective authentication, immutability, and reliability, are met by IoT technology, which is commonly used as a tool in a range of disciplines, including bitcoin and the health care industry [29]. Additionally, a decentralized processing infrastructure is offered, removing the chance of a single point of failure. Regarding blockchain-based IoT solutions, several suggested solutions must reduce and stop DDoS attacks on various IoT device tiers.

The study in research [30] used Ethereum blockchain to mitigate the DDoS attack coming from IoT devices. Each IoT device has a unique IP address needed to connect and communicate with the target network or server; the proposed solution used Ethereum private blockchain and a smart contract to check the IP address of each device to determine if it was legitimate or not. If legitimate, the IP address was authorized and allowed to enter the network and communicate with the server; if not, the blockchain denied it entry to the network and communication with the server. The proposed approach used a private blockchain with a centralized nature science where only one node was responsible for the verification process. This paper did not mention how the blockchain performs the trusted process. In addition, the authors failed to specify which DDoS and IoT layer they concentrated on.

Alternatively [11] suggested a blockchain architecture that integrates the IoT to stop DDoS attacks from IoT devices. The design makes use of both smart contracts and the Ethereum blockchain. First, IoT devices must register with the servers to transmit and receive messages to/from other IoT devices. IoT devices can only function up to the predetermined gas limit before ceasing to function. A server may unregister or remove any IoT device that experiences network failure or whose gas limit has run out.

Additionally, the server is in charge of creating and registering smart contracts. The server sends the registered contract’s address to all IoT devices in the network. An IoT device registered with the server is added to the trusted list of the contract. The gas limit for individual transactions in the contract is defined during smart contract initialization to protect against DDoS attacks. The smart contract is the key regulator that wants to focus on all the participating IoT devices. It not only allows but also restricts the use of IoT devices up to the gas limit. An IoT device contacts a smart contract to transmit a message; if the IoT node’s address is found in the trusted node list, the message is recorded on the blockchain; otherwise, the message is dropped. This architecture has the idea of keeping a trusted list on the smart contract that is checked every time a new message is sent by a device or interacts with another device. The authentication process is completed after the IoT device’s address is stored in the trusted list on the smart contract. As a result, scalability difficulties will always exist in such a system. The process of trusting the IoT device at the server during registration has not been discussed in depth.

The study in research [9] used a blockchain IoT (BCIoT) framework; this framework was used as a decentralized solution to solve the security issues that exist in the centralized solution, so they proposed to use end-to-end security based on the blockchain and smart contract to provide a secured communicative environment by using a hash-based secret key for encryption and decryption processes. This decentralized solution is all controlled, and the core data is stored on the blockchain. As a result of this study, the availability is increased. The fundamental drawback of this framework is that all data, whether it originates from a malicious device or not, is stored on the blockchain, which causes scalability problems.

The authors of [30] developed a blockchain-based framework for a multilevel DDoS mitigation technique (ML-DDoS) to defend IoT devices and other computational resources or machines. The main idea behind the suggested method is to utilize a device-based verification system based on blockchain to keep malicious devices out of IoT environments. The proposed framework’s performance was assessed using three benchmark apps and was constructed using the blockchain-benchmarking tool Hyperledger Caliper. The findings demonstrated that the suggested framework improves throughput by up to 35%, delay by up to 40%, and CPU utilization by up to 25%.

Numerous research papers have been published on lessening and preventing the Mirai attack. For example [7] suggested using blockchain technology to safeguard IoT devices from Mirai botnet attacks. The host is established in one of the proposed network’s various Autonomous Systems (AS). The list of internet protocol (IP) addresses for each host or device connected to AS is utilized in this case to store and communicate with other nodes to identify hosts that have harmful software. Every AS watches the communication process in this network and compares the number of packets created by each host with the threshold value to determine whether that host or device has malware. The authors asserted that, by using their simulation to ascertain the precise value for the malicious threshold, they do not alter the response time on the victim target because the proposed approach efficiently blocks the malicious packet from the infected host. They used Java to create their simulation. They successfully mitigated the Mirai botnet assault due to their detection, which had a 95% true detection rate when the harmful detection threshold value was 8. One node is in charge of the verification process in this paper’s private blockchain, which has a scientifically centralized nature.

In [31], the authors primarily focused on real-time packet-filtering mechanisms to ensure that only legitimate users can access the service. The Ethereum blockchain platform and NS3 simulator were used to simulate their model. As opposed to the actual packets received at the source, it was demonstrated that the traffic is substantially decreased by at least 10%. The packet-marking approach theory has also demonstrated that, after removing the unknown flooding of packets, the incoming traffic to the Blockchain network represents 90% of the proper traffic flow. In the same context, researchers in [32] proposed a proactive IoT botnet detection system that looks for unusual IoT device behavior and minimizes DDoS botnet exploitation at the source end. Discovered bots are also prevented from infecting other IoT devices through a collaborative trust-relationship-based threat-information-sharing approach. The researchers used Hyperledger and the Ethereum Virtual Machine to evaluate how collaborative threat intelligence is shared. The proposed approach can identify 97% of the Mirai botnet attack activity.

To increase security and accessibility, several works were combined with blockchain technology, including [12]. To protect IoT devices from various cyber security assaults, including DDoS, the authors of this article recommended integrating DDoS threat signaling with blockchain technology using Ethereum and smart contracts. These values enable testing using threshold calculation against the variation of humidity, pressure, temperature, and wind direction on that day to determine whether an IoT sensor is under a DDoS attack. The datasets used for evaluating the resulting process contained data from four sensors over two months. These findings demonstrated the DOTS’ ability to assist attack detection when used for IoT edge computing. There was no discussion of the authentication and implementation processes, which is this paper’s biggest drawback.

In [33], a deep-learning-based blockchain system was proposed, where switch authenticity is managed by the blockchain and a deep Boltzmann machine conducts anomaly detection. Using the zero-knowledge proof idea, each switch is registered on the blockchain and confirmed using consensus techniques. The deployment of deep-learning-based models allows the identification of DDoS attack characteristics over the network. An emulator for mini-nets was used to evaluate the framework. The KDD dataset was used to train the deep learning model for network system anomaly detection. The study indicated a 5–10% increase in detection effectiveness, but at a cost that was comparatively higher than that of previous models.

Although [34,35] made the integration between blockchain and Software-defined Networks (SDN), in [36], the authors proposed the Co-IoT framework, which is a blockchain framework using Ethereum smart contracts and collaboration with Software-defined Networks (SDN) to mitigate DDoS attacks on IoT technology. The authors claimed that this framework offers decentralization, secure collaboration among multiple SDN-based domains, efficiency, flexibility, and cost-effectiveness, making it a good scheme to mitigate DDoS attacks on a large scale. They evaluated the performance of this framework in terms of (flexibility, efficiency, security, and (cost-effectiveness)).

The authors of [35] developed a novel, adaptable model to mitigate DDoS assaults. In addition to the current DDoS mitigation measures, which provide public and distribution infrastructure to establish the white- and blacklists for the IP addresses across various domains, blockchain technology and smart contracts must be leveraged. Additionally, this technology must be integrated into the current DDoS defensive system without developing new, highly customized distributed systems. The writers of this solution proposal employed Software-defined Networking (SDN), which offers a practical method for enabling dynamically adjusted rules and services.

BlockSDSec used blockchain as a service for SDN DDoS mitigation. The SDN framework establishes communication between the controller and switches using the OpenFlow (OF) protocol. The key concept is employing blockchain on the OF switches to protect data integrity from tampering caused by a DDoS attack when corresponding with the controller. Additionally, data from every layer of the SDN is added to the block, assuring validity and integrity. The deployment is the only thing covered in detail throughout the experiment. In particular, DDoS resilience has not been verified for the configuration [13].

The authors of [36] proposed using blockchain and SDN to reduce DDoS attacks. Any business can use this network design as a fully loaded DDoS mitigation security solution. The proposed design of this architecture is extremely flexible and can be used to mitigate assaults in various areas. The ability of SDN to correctly authenticate and filter traffic offers a good method for verifying real persons. Regarding Blockchain, its powerful consensus mechanism offers a solution for storing the trusted IP address trust list.

The authors in [37] also proposed a blockchain-based SDN-targeted DDoS defense system (BSD-Guard). It can offer SDN controllers a cooperative detection and mitigation method. BSD-Guard adds a secure intermediate plane based on a blockchain between the control and data planes. The secure middle layer determines the suspect rate of incoming flows based on the information from the gathered packets. It sends suspect lists to the blockchain for immutable storing and sharing. The examination results showed that BSD-Guard could accurately issue defensive strategies close to the source of attack by identifying the attack path and detecting DoS/DDoS attacks with many controllers.

The authors of [10] secured configuration files from DDoS attacks using blockchain extension and smart contracts. Blockchain successfully protects the transaction records in fog networks, and a blockchain-based network configuration thwarts attempts to tamper with transactional data. Table 1 summarizes the related works of blockchain-based decentralized solutions, outlining the benefits and drawbacks of each study.

A public blockchain can be utilized with this system to obtain all the decentralized blockchain qualities, in contrast to the research stated above, which did not implement the suggested model in practice. Additionally, how IoT devices are authenticated before and after being stored in the blockchain is detailed. According to [9], the proposed system only requires the IP address to be stored inside the blockchain; if the device is authentic for communication with the server, the data is stored. This contrasts with existing systems that require all IoT device data to be stored in blockchain before verifying the device’s authenticity.

## 4. Proposed Prevention System

We implemented a decentralized authentication approach using blockchain technology to prevent DDoS attacks on the application layer in IoT technology.

### 4.1. System Overview

With the public Ethereum blockchain and smart contracts, this system suggests a way to authenticate any device before adding it to the IoT network. Considering the following aspects, we decide to use the Ethereum blockchain technology. The first is that Ethereum is one of the most powerful decentralized blockchain platforms and has one of the highest levels of resistance to data falsification and cyber-attacks. The second aspect is that it offers secure transactions using the Elliptic Curves Digital Signature Algorithm (ECDSA) with a key length of ecp256k1. This digital signature is a robust and lightweight signature method for limited devices such as IoT devices. Moreover, the latest version upgrade—released in December 2020 (24 April 2022)—improved the Ethereum network scalability issues and reduced the delays by increasing the number of transactions for the network.

The need for such prevention arises from the fact that, if this layer is hacked, the entire application could suffer flaws that could stop and block all services, making the application no longer functional. More thoroughly, our proposed system has the following functionalities:

- It allows the verifier to identify manager IoT devices that can create groups with different unique IDs.
- It has the ability to compile and deploy a smart contract by the verifier or developer on EVM to generate the address we chose for mandatory use to run all system functions.
- It allows the verifier to add a follower IoT device based on an existing group and ensures that the follower already has an identity card assigned by the manager IoT device before joining the blockchain.
- It prevents follower IoT devices from communicating with the target server if the object’s ID does not exist on the white trusted list or if it exists on the white trusted list. Still, it exceeds the defined factor value called (gas limit).

### 4.2. System Design

As previously mentioned, the system is built utilizing the Ethereum blockchain and the smart contract, an executable program written by a verifier. Additionally, because Ethereum is a public open-source blockchain, other users have access to it. The following are the main components of the system:

- Verifier or Developer: who implements the smart contract.
- Manager IoT Device: who creates the group and assigns the follower IoT device’s lightweight certificate using the manager IoT private key.
- Follower IoT Device: who sends request transactions to join the blockchain and then sends another transaction to communicate with the main server on the IoT network.

#### 4.2.1. Initialization Process

At this phase, the Verifier needs to create and compile a smart contract, which is the main part containing all our system functions, by entering the device address and port. However, before entering the IP address and the port, we run the Ganache CLI, an updated version of Test RPC. Ganache CLI is a tool designed to be a blockchain emulator (virtual environment) that can be used as an alternative to running an actual Ethereum environment. After pressing a “compile smart contract” button, the application creates a smart contract and deploys it as a new transaction in the blockchain. Then, the contract address is returned to the system.

Moving forward, an IoT device can define itself as a manager device or follower device based on its choice. If it defines itself as a manager with public and private keys, then it can create a group with a unique group ID. However, suppose it is selected as a follower device; in that case, it generates Elliptic Curve (EC) public and private keys, which provide the identity card, a lightweight certificate containing the follower’s public address, group ID, follower object ID, and the signature of the manager’s private key, using ECDSA.

Successful group creation is established after checking the group ID’s existence and the manager ID’s uniqueness. If landed successfully, any follower device with a valid identity card can join only one specific group. After that, the follower sends a registration request to the verifier, and then the verifier’s smart contract can verify the uniqueness of the follower object ID alongside the follower ticket using the public key of the master manager. If this process is done successfully, the follower IoT device can join the blockchain, and the smart contract stores the object ID and IP address inside the white trusted list.

#### 4.2.2. Communication Process

This process is conducted after successfully adding the follower device to a specific group. The purpose of the group ID for each follower is to prevent any IoT devices with no group ID from joining and communicating with the target server, which is the first step in preventing any malicious device from joining the IoT network. The system allows the follower IoT device to communicate with the target server after satisfying two conditions:

- Validating if the follower IoT device object ID is already stored in a white trusted list that contains only the authenticated follower devices.
- Checking the gas limit value specified in a smart contract. The gas limit value refers to the fee required to conduct a transaction on Ethereum successfully. Each device has a different gas limit value due to the nature of the entire transaction. Therefore, if any device exceeds the gas limit value, it will identify the target server as a DoS attack. The technical inference here is that we should monitor the device’s behavior. If we notice any device with abnormal behavior, then this device must be labeled and classified as a malicious device. From here, the system will drop it from the white trusted list.

**Figure 2:**
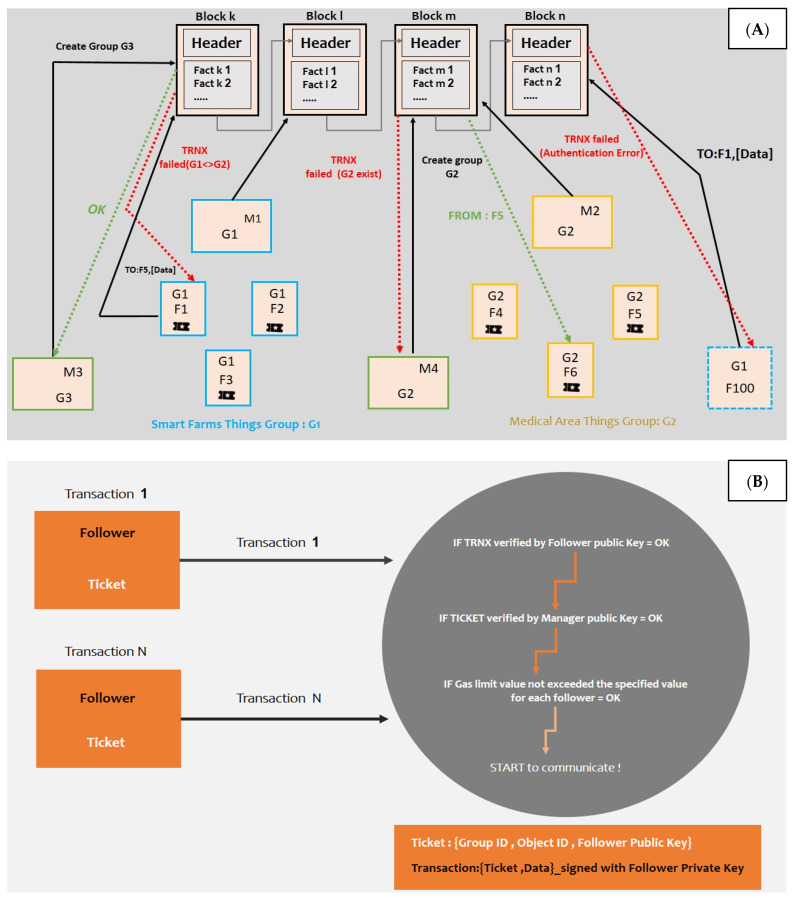
(**A**) Initialization phase; (**B**) Communication phase.

Figure 2 illustrates the proposed system: (A) demonstrates how the initialization phase is conducted, and (B) shows the communication phase with the server. Specifically, Figure 2A highlights how the blockchain makes access control upon the objects and transactions. For example: (1) unlike Manager M2, who can create the group G2, Manager M3 cannot create the group G2 because it already exists. (2) Unlike the accepted message exchanged from F1 to F2, which belongs to its group G1, the exchanged message from the object F5, belonging to the group G2, to the object F1, belonging to the group G1, is rejected by the blockchain. Figure 2B demonstrates how communication transaction occurs after two checks are made: First, of the validity of the follower IoT device’s identity card using the manager IoT device’s public key, and second, of the IoT follower device’s gas limit value; this enables the IoT device to communicate with other IoT devices on the same network if these checks are successful.

#### 4.2.3. System Setup and Requirements

The system can now be constructed by setting up and installing the following environment and libraries after the two primary processes have been established:

##### Environments

The following environments are needed to build the proposed system:

- Remix IDE: a development environment and open-source web and desktop application. It comes with a large number of plugins, and it has a user-friendly interface that supports a quick development cycle. Furthermore, Remix IDE is generally used for the full smart contract development process.
- Ganache Command Line: a blockchain emulator or local Ethereum client that is quick and easy to customize. It enables the user to make blockchain calls without paying the price of hosting an Ethereum node.
- QT Framework: The Qt is a modern framework with an IDE that comes with many extremely intuitive and modularized C++ library classes and APIs to make application development easier.

##### Libraries and Languages

- JSON-RPC Library: It is a JavaScript library that interacts with the Ethereum blockchain and smart contract functions.
- Solc-js is a JavaScript binding for the solidity compiler that runs via node.js.
- The C and C++ languages.

#### 4.2.4. Smart Contract for the Proposed System

The main component of the dissertation methodology is the smart contract. It is the section that has the functions to implement the system. Smart contract code has these main components:

- Mapping is the key-value type used to store and retrieve values for a specific key.
- Constructor: this is the function to initialize the instance variables used in the smart contract code.
- The modifier is the keyword in solidity language used to create the customized logic. There are two customized modifiers in the smart contract we create. First is the “ControlOf” modifier, which is added in the header of the “Send” function. This ensures the communication process is done only between the IP addresses stored within the trusted white-list. Second, the “OnlyConcernedObject” modifier is added in the header of the “ReadMSG” function; the sole function of this is to assure that the message is readable only by the specified addresses.
- Functions are a piece of code used to deliver the needed requirements. The smart contract has six main functions, BC_Send, BC_ReadMSG, BC_AddNode, BC_SaveNode, BC_Verify, and BC_CheckGasValue. All these functions are divided into two categories, functions related to the initialization phase, such as BC_AddNode, BC_SaveNode, and BC_Verify, and functions related to the communication phase, such as BC_CheckGasValue, BC_Send, and BC_ReadMSG, as shown in Algorithms 1 and 2.

As shown in Algorithm 1, the algorithm specifies the initial phase in our system. In this phase, the contract checks if the group id, object id, and public address for both manager and follower IoT devices are unique or not. If they are not, it will generate an error message, while if they are shown as unique, it will add and save the new node in the blockchain.
**Algorithm 1: Smart Contract Initialization Phase** ***begin***
  ***if***
*(ObjIdExists (obj.id, bc) == true)*
***then***
   ***return***
*Error ();*
  ***if***
*AddrIdExists (obj.grpId, bc)*
***then***
   ***return***
*Error ();*
  ***if***
*(obj.type == manager)*
***then***
  ***{***
   ***if***
*GrpIdExists(obj.grpId, bc) == true*
***then***
    ***return***
*Error ();*
  ***}else if***
*(obj.type == follower)*
***then***
   ***{***
    ***if***
*GrpIdExists(obj.grpId, bc) == true*
***then***
    ***return***
*Error ();*
   ***}if***
*(bc.CertificateVerif (obj.certificate) == false)*
***then***
    ***return***
*Error ();*
   ***else***
    ***return***
*Error ();*
  ***end***
*// Initialization phase finished with success*

Moreover, Algorithm 2 specifies the communication process between the IoT device and the target server. The communication process is unsuccessful if the IP address of the IoT device does not appear on the trusted white-list or does so but exceeds the gas limit amount. On the other side, the communication process is successful if it is done the other way around.
**Algorithm 2: Smart Contract Communication Phase*****begin***
  ***if***
*(ObjIdExists (sender.id, bc) == false OR ObjIdExists (receiver.id, bc)== false)*
   ***then***
    ***return***
*Error ();*
  ***if***
*(sender.grpId != receiver.grpId)*
***then***
    ***return***
*Error ();*
  ***if***
*(bc.SignVerif (sender.msg) == false)*
***then***
    ***return***
*Error ();*
  ***if***
*(bc.CurrentGaslimitValue >(AllowedGasLimitValue))*
***then***
    ***return***
*Error ();*
    *LabelDeviceAsMalicious();*
    *dropFromWhiteList();*
  ***end***
*// Communication phase finished with success*


## 5. Results and Evaluation

This section demonstrates the system’s evaluation and testing. The existing centralized solutions also allow the research to evaluate the security requirements of the proposed system. The dissertation covers the main functions’ time complexity and code efficiency.

### 5.1. System Evaluation

The system can be evaluated based on different aspects and factors; we focused on security and its issues and the system’s performance when it performs the entire task. These combined aspects and factors allow users and clients to activate the functionalities. Performance and cost are mainly measured by time; any successful system should perform the tasks correctly with the best and most optimal way to utilize the system resources efficiently and effectively. As such, this chapter shows the proposed system algorithms and each algorithm’s time complexity, representing the main algorithms using the pseudo-code technique.

#### 5.1.1. Comparing Existing Centralized Solutions and Our Decentralized Solution

The solution is based on a decentralized methodology. User flexibility is the main aspect that makes decentralized systems superior to centralized ones. Users in a decentralized system have full control of their transactions. They can start their transactions and communicate without authorization from a centralized party. A decentralized system does not have a central authority that governs the whole network. This gives the users high control and security due to the immutable data. No one can modify anything illegally, making decentralized systems more secure than centralized systems. In addition, a decentralized system does not contain a single point of failure, while centralized systems suffer from a single point of failure. Centralized systems have limitations in scalability, but a decentralized system is scalable without any limitation.

#### 5.1.2. System Security and Integrity

As mentioned before, decentralized systems have high security, and the following paragraphs describe how the proposed solution satisfies important security principles.

- **Authentication and message integrity:** A certificate is used by each IoT device’s followers (for the initialization transaction). During the initialization process, the certificate is only sent to valid objects. All exchanged transactions are signed using the private keys associated with the certificates using the ECDSA algorithm. As a result, signatures protect the device’s authenticity and the message’s integrity, which ensures that no device can join the network without the certification.
- **Identification:** Each IoT Device has a unique identification (an object ID linked to a group ID and a public address). The Manager’s signature on the certificate ensures that this identity is trustworthy. This device’s private key, tied to its identification, is used to sign each message it sends. As a result, the system can recognize it immediately.
- **Non-repudiation:** Since the transactions are signed with a private key only known by the device that owns it, it can only be used by that device. As a result, it cannot deny that a message was signed.
- **Scalability:** Our system is constructed on a public blockchain, built on a peer-to-peer network. Peer-to-peer networks are well recognized as one of the greatest solutions for a large-scale system.
- **Reliability:** Our decentralized system is dependable because it uses a P2P network that is considered dependable according to the entire function. If a part of the system fails, other parts are not affected, and they will still be running; then, the decentralized system is not a single point of failure.
- **Sybil attack protection:** In our system, each device can only have one identity at a time, and each identity can only have one key pair. The private key associated with this identity must sign each communication message. Furthermore, the system must authorize all IDs, so an attacker cannot use a false identity.
- **Message replay protection:** Every message is recognized as a transaction. Each transaction has a timestamp and must go through a consensus phase to be considered legitimate. As a result, an attacker will be unable to respond to messages since the consensus process will reject them.
- **DoS/DDoS protection:** Blockchains are robust to DoS/DDoS cyber-attacks due to their decentralized architecture. Services are indeed copied and spread over multiple network nodes. That is to say, even if an attacker gets to disable one node, he or she will not be able to disable all of the other nodes. Furthermore, transactions are expensive, discouraging an attacker from sending a large number of transactions. Furthermore, in some blockchains, such as Ethereum, the price of a transaction is linked to the amount of data sent.

#### 5.1.3. Evaluating System Performance

The system should use the resources efficiently. Modern systems not only focus on building a system that functions correctly but also on how much time each function needs to perform a specific task. Time is the most important factor in whether a system’s performance is good or bad. We can use the standard big O notations to measure a time-consuming function or algorithm. The system that takes constant time is better than another system that takes linear time, linear time is better than logarithmic time, and logarithmic time is better than quadratic or cubic.

##### Algorithms and Time Complexity

In this section, we will evaluate the main functions and algorithms and compute the time complexity of them as in the following:

- **Verify Node Algorithm Pseudo Code (Algorithm 3):** this algorithm is used to check if the follower IoT device has a card identity or not; it takes a time complexity of ***O***(***C***), where *C* is constant. This algorithm will only add a constant effect to the complexity.

**Algorithm 3: Verify Node Before Joining it Into Network*****//This code checks if a node has a card identity*** ***//It is a Boolean returning value that returns true if the node has a card or returns false when the node does not have a card*** ***Begin*** ***if ((ecrecover(hash, v, _r, _s) == masterAddr) || (ecrecover(hash, v + 1, _r, _s) ==masterAddr))***
   ***then***
    ***Return true;***
   ***else***
   ***Return false.***
   **End;**

- **Convert From Address to Byte Algorithm Pseudo Code (Algorithm 4):** this algorithm is used to convert the hexadecimal address to bytes (*n*) to be easily used in the computer system. This algorithm will take a time complexity of ***O***(***n***) because of the loop iterations, so it has a linear time complexity.

**Algorithm 4: Convert from address to byte**

 ***Begin***
  ***bytes memory baddr = FromAddressToBytes(addr); // Call Method that convert***
    ***bytes memory res = new bytes (1 + 1 + 20);***
   ***uint i = 0;***
    ***res[i++] = byte(v1);***
    ***res[i++] = byte(v2);***
    ***uint j = 0;***
    ***for (j = 0; j < n; j++)***
  ***{***
     ***res[i++] = baddr[j];***

  ***}***
      ***return res;***

  ***End;***

- **Add Node Algorithm Pseudo Code (Algorithm 5):** This algorithm is used to add the new follower IoT device (*n*) inside a group (*m*) in the blockchain network. The time complexity (worst case) will be a time complexity of ***O***(***m*** + ***n***). It will check m conditions for outer nested ifs until finding the true condition and will check n conditions for the inner nested if. The best case is if the condition is matched at first, if that may take constant time as in a simple if-else statement.

**Algorithm 5: Add Node to Network**
***//this function take important parameters such as category, group Id, Object id*** ***Begin*** ***if (ids[msg.sender]. length != 0)*** ***Return error();***
   ***if (NodeMemberId != address(0))***

***Return error();***
   ***if (_category == 0) {***
    ***if (grpIdMasters[_grpId] != address(0))***

***Return error();***
    ***else {***
    ***grpIdMasters [_grpId] = msg.sender;***
    ***}***
   ***} else {if (CallVerifyFunction == false) {***

***Return error();}***
***else { Call SaveNode(msg.sender, _grpId, _id) function;***
***} }***
   ***End;***

- **Communications between Nodes (Send/Read Messages by Destination) (Algorithm 6):** This algorithm is used to establish the communication process (Read and Send) between IoT devices in the same group; it will take a time complexity of a constant time ***O***(***C***), where *C* is constant, and a good network configuration will improve sending and receiving messages.

**Algorithm 6: Communications between Nodes (Send and Read Messages by Destination)**

 ***Begin***

  ***Send (sender, receiver, string memory msg)public ControlOf(sender, receiver) //***   ***send message {***
     ***boxes[receiver] = msg; }***
  ***ReadMSG (addr) OnlyConcernedObject(addr)public returns (string memory) //***
 
  ***Read message by intended destination***
   ***{ return boxes[addr];***
   ***}***

  ***End;***

- **Save Node Algorithm Pseudo Code (Algorithm 7):** This algorithm is used to save the IP address of the follower IoT device inside the white trusted list in the blockchain; it takes a constant time complexity *O*(*C*), where *C* is constant.

**Algorithm 7: Save Added Node**

 ***Begin***

  ***SaveNode(address _addr, _grpId, Object _id) public{***
    ***ids[_addr] = AddWith(_grpId, _id);***
   ***}***

  ***End;***

- **Check Gas Limit Value Algorithm Pseudo Code (Algorithm 8):** This algorithm has a nested loop—two for loops that iterate among all element’s nodes(*n*) and check every node’s gas value if it exceeds the maximum given gas value, so the worst and average case will be a time complexity of ***O***(***n***^2^). The best-case scenario is when the algorithm finds the node that exceeds the gas limit in the first location or index in the array; there is no need to iterate all loop iterations, and the time complexity will decrease.

**Algorithm 8: Check Gas Limit Value**

 ***Begin***

***Arr[allNodes];***

***Define MaximumGasValueGiven = Arr[NodeID].GasMaximum;***

***For i to n {***

***For j = i + 1 to n***

***{***

  ***If(Arr[j].GasValue > MaximumGasValueGiven)***

***Return Error();***

  ***LabelDeviceAsMalicious();***

  ***DropFromWhiteList();//Delete element from array***

***} }***


Only one function in a worst-case scenario may take a quadratic time complexity. If we improve it, it may get a logarithmic time complexity in some cases, which will be better than quadratic. Most functions constantly take a linear time complexity, meaning the algorithms and code are written optimally to improve system performance as much as possible.

##### Evaluation Results

This section will discuss the results of applying the proposed system using SolarWinds software and an online CPU stress test and utilization. We used two separate machines with different processor and RAM specifications, as shown in Table 2.

Additionally, a description of the parameters of the modeling and simulation environment with which we are concerned when testing the proposed system can be found in Table 3.

**Figure 3:**
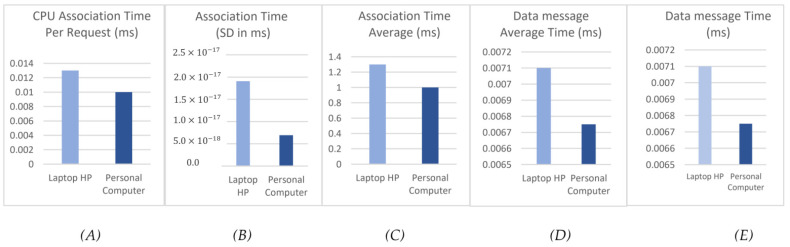
Evaluation results: (**A**) CPU Association Time Per Request (ms), (**B**) Association Time (SD in ms), (**C**) Association Time Average (ms), (**D**) Data message Average Time (ms), and (**E**) Data message Time (ms).

After performing 100 experiments, the assessment findings in Figure 3 demonstrate the following: The first Figure 3A represents the time required for a follower to make an association request. Each IoT follower device takes around 0.013 ms using the HP laptop machine and 0.010 ms using a PC machine to perform an association request to join the blockchain. In the second Figure 3B, the system calculates the standard deviation (SD) to give a deep analysis of how much variation there is in the values. Moving forward, the system has a low SD value in both machines, which indicates a stable and consistent performance of the proposed system. The third Figure 3C represents the average association time for 100 experimentations, which equals 1.3 ms using an HP laptop machine and 1.0 using a PC machine. In the last two Figure 3D,E, the system calculates the time required to transmit the message; the system has 0.0071 ms per message using an HP laptop machine, and the average needed time is 0.71 ms. Using a PC machine, each message needs 0.00675 ms for transmission and 0.675 ms as the average time for all messages.

Additionally, we have calculated the Big O notation for each time factor in Figure 3. Big O notation is a mathematical notation to measure a time-consuming function or algorithm when the argument tends towards a particular value or infinity. For instance, CPU Association Time Per Request (Figure 3A) will take a time complexity of O(n+m) where *n* is the IoT device, and *m* is a group in the blockchain network. When it comes to Data message Time (Figure 3D), it will have a time complexity of O(C) where *C* is the constant value.

##### Comparing the Results with Related Works

Table 4 compares the results with the existing state of the art based on the authentication time. For instance, the authors of [26] offered an authentication system based on a Datagram Transport Layer Security (DTLS) algorithm. The association phase (DTLS handshake) in the DTLS requires at least five messages. Other messages, such as the Change Cipher Suite Message (CCSM), can also be added. Finally, up to eight messages in the association phase require a time of 21 ms per request. Additionally, the authors in [27] developed an Elliptic-Curves-Cryptography (ECC)-based authentication system for Wireless Sensor Networks (WSN), which needs five messages for the association phase, requiring a time of 23 ms per request. In addition, it needs to use a gateway, which can double the number of user messages. Other systems like [38] suggested a more robust authentication scheme, which needs at least four messages in the association phase. It is important to highlight that the I/O operations are considered the most expensive and time-consuming (as evidenced by the testing outlined in Figure 3). Any system that has a fewer number of I/O operations is considered to be a more efficient and effective system. Hence, our proposed system highlights two messages: the first is the request transmitted from the IoT follower device to join the blockchain, and the second is the blockchain response with a time of 0.012 ms. The latter takes much less time due to the small number of I/O operations.

Another comparison with previous studies was made based on indicating what service is provided to mitigate or prevent DDoS attacks, in addition to the type of protocol used to implement the method used, as shown in Table 5.

As shown in Table 5, the authors in [7] offer authentication types based on the Autonomous Systems (AS), which have a centralized nature and are used to mitigate DDoS attacks after they occur. Additionally, the authentication process mainly depends on SDN, and the blockchain is just used for attacker record sharing as in other studies [13,33]. The authors in [10] developed a new blockchain expansion algorithm to authenticate the configuration file, which means the blockchain is just used as a record keeper. On the other hand, our proposed system is used a decentralized “Blockchain technology” mainly for the authentication process by using the ECDSA algorithm as the first step before any IoT device joins the network to prevent any malicious device from communicating with the server. In addition, the blockchain is used to record and trace the IP address of malicious devices to share with other IoT devices inside the network.

## 6. Conclusions and Future Directions

In this research study, a system is suggested for preventing DDoS attacks, the primary security issue with IoT technology. The main aim of this study is to replace existing centralized system solutions with a decentralized one to prevent this attack on IoT devices on the application layer by authenticating and verifying these devices using public blockchain technology, which provides an authentic and tamper-proof platform. Additionally, this study shows how the IoT devices at the blockchain level verify and authenticate using a trusted white-list implemented in the smart contract. In addition, this decentralized solution has no hardware upgrade for IoT devices because the design can be built as an overlay network on top of the existing conventional network.

This study mainly focuses on preventing DDoS attacks only on the application layer of IoT technology. In future work, the proposed system will be developed to mitigate and prevent this attack on the network layer from providing more security in IoT technology. Additionally, this system is implemented using a public blockchain with a scalability limitation problem. In the future, the system will eventually address this issue by putting forth various options and comparing them to choose one that works best. Furthermore, blockchain can be employed for data analytics applications and cyber-attacks in communicating data, such as data compressing algorithms for transactive energy management framework [39] and for healthcare security analytics [40].

## Figures and Tables

**Table 1:** Blockchain solutions (advantages and disadvantages).

Study	Advantages	Disadvantages
Ahmed et. al. [7]	Blockchain technology stores and shares the list of IPs for each device with other nodes by comparing the number of packets each device generates with the threshold value. This mitigates the Mirai attack.	The type of blockchain is private and has a centralized nature. Only one node is responsible for the verification process.
Natsheh et al. [8]	The Ethereum blockchain is used, and the legitimate list is created using a smart contract to check if the device’s IP address is legitimate or not to communicate with the server.	The proposed solution used a private blockchain with a centralized verification procedure that only one node can manage. The authors fail to specify the DDoS kind and IoT layer they are concentrating on.
Badruddoja et al. [12]	Blockchain technology is integrated with DOTS to help to detect the DDoS attack when mapped on IoT edge computing.	The authentication and implementation processes are not discussed in depth.
Javid et al. [11]	A blockchain with IoT integration and a predetermined value called the “gas-limit” are utilized; if the device exceeds this value, communication with the server is prevented.	Scalability difficulties will always exist in such a system. During registration, the process for trusting an IoT device at the server is not discussed in depth.
Jamader et al. [9]	The framework (BcIoT) utilizes end-to-end security based on the blockchain and smart contracts to create a safe communication environment and improve availability.	The fundamental drawback of this approach is that all data, whether it came from malicious software or not, is stored on a blockchain, which causes scalability problems.
Bose. et al. [13]	Blockchain can ensure the quality and integrity of data traversing between SDN layers.	There is no experimental evidence for DDoS mitigation. The only thing implemented is the setup. The experimental method and results are not presented clearly.
Gul. et al. [10]	Blockchain successfully protects the transaction data in fog networks. Blockchain-based networks prevent any attempt to change transactional data.	Blockchain is only used as a record-keeping system.
Singh et al. [33]	The blockchain secures switch registration and verification, while a deep Boltzmann machine helps anomaly detection. The effectiveness of detection is increased significantly.	The cost of computation and communication is higher.

**Table 2:** Machine Specification.

Machine Name	CPU Architecture	CPU Mode	CPU Processor	RAM	OS
Laptop HP	Intel(R) Core(TM) i7	x64-based	CPU@2.40 GHz 2.40 GHz	8 GB	Ubuntu 22.04
Personal Computer	Intel(R) Core(TM) i7	x64-based	CPU@2.60 2.60 GHz	16 GB	Ubuntu 22.04

**Table 3:** The selective parameters of the modeling and simulation environment.

Parameter	Parameter Responsibility
**Initialization Phase**:	
1. Unique manager device ID and unique group ID.	These parameters are needed to create a new group inside the blockchain, such as (the smart home group).
2. Follower device public address, follower group ID, follower object ID, and the signature of the manager’s private key.	These parameters are needed to create the follower identity card or certificate as a first step to joining the blockchain by signing with the manager’s private key.
3. Follower device private key and manager public key.	ECC digital signature algorithm is used to check the validity of the follower identity card or certificate using the follower private key before joining the blockchain by verifying it using the manager public key.
**Communication Phase:**	
1. Follower device object ID and trusted white-list	These are needed to validate if the follower IoT device object ID is already stored in a white trusted list that contains only the authenticated follower devices.
2. Gas limit value	Each device has a different gas limit value due to the nature of the entire transaction. Therefore, if any device exceeds the gas limit value, it will identify the target server as a DoS attack.

**Table 4:** Comparison of the results with related works based on the authentication time.

Related Work	Protocol/Number of Messages	Authentication Time per Request (ms)
[26]	DTLS/8 Messages	2.1 × 10^1^ ms
[27]	ECC/5 Messages	2.3 × 10^1^ ms
[38]	NA/4 Messages	>1 ms
Proposed System	ECC/2 Messages	1.2 × 10^−2^ ms

**Table 5:** Comparison of the results with related works based on the service and type of authentication protocol.

Reference/Year	Service	Type of Authentication Protocol	DDoS Mitigation/Prevention
[10]/2020	Blockchain and smart contracts are used for securing configuration files and transaction records in a fog network against DDOS attacks.	The blockchain Expansion Algorithm is used to determine if connections may be created between databases of other parties and the fog. Once a special-duty fog node has been discovered, it offers cloud services that allow connectivity between clouds without needing an additional connection.	Prevention.
[13]/2019	Blockchain is used to mitigate DDOS attacks in SDNs by ensuring data transfer between their layers’ integrity and validity.	The virtual controller verifies the transactions or flows table entries. When it discovers the correct flow entries in the legitimate switches, the actual SDN controller updates the entries in the same way.	Mitigation.
[33]/2021	Deep learning and blockchain avoid DDOS attacks in SDN industrial networks.	A blockchain using a voting-based consensus mechanism and a deep-Boltzmann-machine-based flow analyzer are deployed at the control plane to authenticate the anomalous switch requests.	Prevention.
[7]/2019	SDN, blockchain, and smart contracts mitigate the Mirai botnet attack.	The task of forwarding packets from connected hosts outside the network falls under the Autonomous System’s (AS) responsibility. The AS is responsible for maintaining a list of host IP addresses and the threshold for each host that determines whether the host is malicious or not.	Mitigation.
This Work/2022	Ethereum blockchain technology and smart contract are used to authenticate and validate IoT devices, which provides an authentic and tamper-proof platform to replace the present centralized system solutions and prevent DDoS attacks on IoT devices on the application layer. Additionally, to prevent connecting to and communicating with IoT networks in the future, we track and store the IP addresses of malicious devices inside the blockchain.	The ECDSA algorithm is used to check the validity of the follower identity card or certificate using the follower private key before joining the blockchain by verifying it using the manager public key to prevent any malicious device from communicating with the server.	Prevention.
